# Cinchonidia Sulphate

**Published:** 1876-06

**Authors:** E. H. Purdum

**Affiliations:** Hermon, Ill.


### CINCHONIDIA SULPHATE.

By E. H. PURDUM, M.D., Hermon, Ill.

(Read before the Military Tract Medical Society, at Galesburg, Ill., Jan. 11, 1876.)

In the early part of last year (1875) my attention was called to cinchonidia sulphate, and since that time, I have used it quite extensively, especially in intermittent and remittent fevers, and with results equal, so far as I could see, to those obtained by the use of sulphate of quinine; and with this advantage: my patients make no complaint about the disagreeable noises in the head, which accompany the use of quinine in the large majority of cases, though they generally supposed they were taking the latter drug. The dose I aimed to have correspond with those of the quinine, when given under similar conditions.

The irritation of the stomach does not exceed that of any other of the alkaloids.

In illustration of its virtues I subjoin a few cases, or rather a synopsis of them:

Case I.—Was called, Aug. 6th, 1875, to see G. L., male, aged 18. Has had general good health until within the last few days. On the 5th day preceding my visit, he had some slight chilliness alternating with hot flashes; feels sore all over; has no appetite; bowels have been constipated for several days; tongue enlarged and coated heavily; pain in the head, and slight tenderness over the region of the liver; and there is some fever.

The residence of this patient is on the northwest side of Cedar creek, in the bottom, and not over eighty rods from it, surrounded on three sides by high hills, and with the prevailing winds from the south—a good location for malarial fever certainly. I gave him a purgative of comp. cathartic pills. I also ordered 4½ grains of cinchonidia sulphate and two grains each of pulv. Dover and pulv. Zingiber, every four hours.

Aug. 8th.—The patient is much better; has not had any symptoms of chill or fever. Continued treatment for two days more.

On the 17th of August the young man spoken of above, came to my office to get medicine for five others of his brothers and sisters, being entirely well himself.

The five sick children ranged in age from eight to sixteen, and all were having intermittent fever. I prescribed cinchonidia sulphate, pulv. Dover and Zingiber, in four of these cases. I will simply state that the result was all that could have been expected under any method of treatment, and by afterward following this treatment with an antiperiodic mixture, there was no return of the disease in any of these cases.

Case VI.—July 3rd, was called to see W. P., aged 55; farmer. Found him with a fever, which was very high; this had been preceded by severe chill; tongue was very heavily coated, of dark brownish color; bowels constipated; pulse strong and quick; intense pain in the head, accompanied by considerable stupor. I administered a full cathartic, and gave five grains of cinchonidia sulphate and two grains each of pulv. Dover and pulv. Zingiber, every three hours, commencing when the fever subsided. This treatment was continued over the 4th.

July 5th.—Has not had any chill or fever, and otherwise is better. Treatment continued for two days, and case dismissed.

On Aug. 6th, W. P. had another chill, followed by fever; being about one month from his first attack. It will be well to give here a few of the circumstances surrounding this case. He was living in a very malarious locality, viz.: on the north side of Cedar creek, and had been laying drain tile for the purpose of draining a slough and a pond of standing water, which were on his farm—circumstances and conditions peculiarly liable to produce malarial disease.

At the same time (viz., August 6th,) that Mr. P. had his second attack, there were three of his children taken ill; the oldest, aged 16 years, with typho-malarial fever; the other two, aged 8 and 12 years, were cases of well marked bilious remittent fever, and were treated with cinchonidia sulphate, and their recovery was satisfactory, and not followed by any relapse.

These cases are designedly selected from localities very favorably situated to develop malarial fever, and the result of their treatment shows conclusively that the remedy has a very beneficial effect.

Case IX.—Was called Oct. 3rd to see W. J., aged about 38; farmer and stock raiser; living on the south side of Spoon river, and not over eighty rods from the stream. Has not been feeling well for some time, but still able to attend to his farm and stock; two days ago exerted himself very much in trying to drive some cattle, and on the 2nd he had a chill; has been feverish ever since, though the cool air would make him feel chilly whenever he came in contact with it. His bowels were torpid and he had no appetite; he therefore took a dose of pills, which acted pretty freely on his bowels also, producing a free emesis. He has not rested well through the night, and this morning (3rd) he is nervous and restless, being very easily disturbed; there is slight headache; pulse weak and small. The tongue is coated a dark brown in the centre, and the edges are very red; skin dry. Diagnosis: typho-malarial fever. I gave him five grains of cinchonidia sulphate and two grains each of pulv. Dover and pulv. Zingiber, every four hours. In addition, I left bromide of potassium to be administered as required, to allay nervousness and to promote sleep.

Oct. 4th.—Found him quieter. Continued the treatment. On the 5th he was much improved. There has not been any return of the chill, and the fever is subsiding.

Oct. 6th.—Continued treatment, as he was still satisfactorily progressing.

This treatment was continued six days longer, with an occasional cathartic, the patient making steady improvement.

After this time he was put on a mild tonic, which was kept up for an indefinite time.

In all those innumerable ills, such as slight neuralgias, rheumatism, etc., for which we are called to prescribe daily, I almost invariably use this remedy where I formerly used the quinine, and with favorable results.

